# Health Care Access and Utilization by Nepalese Adults in Connecticut

**DOI:** 10.7759/cureus.4543

**Published:** 2019-04-25

**Authors:** Abhishek Thakur, Srijan Adhikari, Daren Anderson, Richard Feinn

**Affiliations:** 1 Internal Medicine, Frank H. Netter MD School of Medicine, North Haven, USA; 2 Neurosurgery, Frank H. Netter MD School of Medicine, North Haven, USA; 3 Epidemiology and Public Health, Community Health Center, Weitzman Institute, Middletown, USA; 4 Statistics, Frank H. Netter MD School of Medicine, North Haven, USA

**Keywords:** nepal, nepalese adult, nepalese, health access, utilization, connecticut

## Abstract

Introduction

Access to healthcare and the utilization of health services at both the state and national levels are frequent areas of study, specifically in major ethnic populations such as White, Black, and Hispanic/Latino. However, there are few studies assessing healthcare access and utilization in the Nepalese communities in the United States (U.S.), despite the rapidly growing population of Nepalese immigrants.

Methods

To explore this issue, we conducted a questionnaire-based survey of Nepalese adults in Connecticut (CT).

Results

When compared to the reporting of the general CT population, a greater percentage of this survey’s respondents report having trouble accessing necessary care (21.1% vs 11.0% in CT). Despite this, more Nepalese adults report satisfaction with the provider in terms of time spent during the visit (81.8% vs 76.0% in CT) and a consideration of values and beliefs during treatment (86.2% vs 70.0% in CT). In comparison with previous national reporting of the general U.S. population, Nepalese adults in the survey tend to have fewer total health care visits annually (87.1% reporting between zero and three visits vs. 64.7% in the U.S.). They also reported fewer dental visits (60.6% vs 71.0% in the U.S.).

Conclusion

While this novel study is one of the few examining health in the Nepalese population in the U.S., it serves as a foundation for future research in this area. Additionally, the results of the study highlight important disparities that local Nepalese organizations can use to design initiatives to improve this population’s health.

## Introduction

It was only in recent decades that people from Nepal had begun immigrating to the United States. While there were very few Nepalese immigrants in the country prior to the 1990s, the population has since expanded. In 1990, the estimated population was just over 2,500 [1]. According to the U.S. Census Bureau American Community Survey five-year estimate, the population of Nepal-born immigrants in the U.S. has risen from approximately 46,177 in 2010 to 95,270 in 2015 [2]. These numbers exclude immigrants who are undocumented or who have exceeded their authorized stay in the country.

While this population continues to increase, it remains unclear how Nepalese are transitioning into the U.S. health care system. Studies done in Nepal have demonstrated significant limitations in health care access and use in the country. Additionally, the country has limitations in several aspects of care, including mental health services, primarily due to a lack of resources and geographical restrictions [3]. Unfortunately, it is unclear to what extent Nepalese immigrants in the U.S. utilize health care services, primarily in comparison to other ethnic groups.

While there is extensive literature assessing the health care of individuals in Nepal, to the best of our knowledge, no similar studies have been conducted on the Nepalese people who have immigrated to the U.S. Previous health care surveys (such as Connecticut Health Care Survey of 2014 and the National Center for Health Statistics 2015 study) have explored disparities between U.S. and CT populations and populations such as Whites, Blacks, and Hispanics/Latinos [4-5]. However, the question remains: do Nepalese immigrants suffer any disparities in their health care in the U.S.?

Our survey attempts to highlight possible differences in the Nepalese population, specifically in Connecticut, in comparison to other U.S. populations, both at the state and national levels. Such information will provide a wealth of knowledge regarding where gaps exist in their care and how care can be improved for these individuals, possibly through policy change and state-level health care initiatives. This study can be an initial step in the understanding of other Nepalese communities throughout the U.S.

## Materials and methods

Survey development

This study was a cross-sectional qualitative survey of the Nepalese population in Connecticut (CT). A short, paper-based survey consisting of 19 questions was developed after a review of previous studies on health access/utilization. The topics included the availability of health insurance, difficulties in accessing care, postponement of necessary care, cost as a barrier to care, language as a barrier to care, frequency of health visits (including the dental and emergency departments), and satisfaction with provided care and perceived health status. The questionnaire was made available in two languages: English and Nepali. The survey was developed to have a Flesch-Kincaid reading level that is below seventh grade to allow for readability.

Participants

Individuals were required to meet the following criteria in order to participate in the survey:

1. Must be a Connecticut resident for one year or more.

2. Must identify as Nepalese. Participants must self-identify within the survey that they are Nepalese. We define Nepalese as “being born in the country of Nepal.”

3. Must be an adult of 18 years or older.

4. Must verbally consent to participate.

Data collection

The Nepalese Association of Connecticut (NAConn) and Society of Nepalese in America were contacted to identify the scheduled gatherings and meetings held by the associations. These gatherings were chosen as locations for survey administration given the large volume of Nepalese in Connecticut that attended, allowing for convenience in data collection. The questionnaire was shared at a booth during two separate Nepalese community events (in West Hartford, CT, and Branford, CT) in the months of April and May 2017. Approvals to conduct the survey at these locations from both associations were acquired prior to data collection. Collected data were then analyzed and compared to previous studies on the general CT and U.S. population. An intrapopulation analysis was also done based on gender and age grouping. Pearson’s chi-squared tests were used for all statistical analysis.

## Results

The study included 244 participants; of these 112 (45.9%) were male and 132 (54.1%) were female. The mean age was 38.7. The percentage of participants who chose to fill out the Nepali version of the survey was 73.4% (vs. 26.6% in English).

Table 1 shows the perception of care among Nepalese adults and the general Connecticut adult population [4]. More Nepalese adults reported experiencing a time that they were unable to get necessary care in the past year (21.1% vs 11.0%). More Nepalese adults reported that enough time was spent with their provider during previous visits (81.8% vs 76.0%). Additionally, more Nepalese adults felt that their provider took into consideration their values, beliefs, and traditions during treatment recommendation (86.2% vs 70.0%).

**Table 1:** Perception of care in Nepalese adults and the general Connecticut adult population CT: Connecticut; asterisk (*): statistical significance (p<0.05)

Response	Nepalese Adults	CT General Adult Population	p-value
Individuals who had experienced a time they could not get the care needed in the past year	21.1%	11.0%	0.000*
Individuals reporting their health status as poor/fair	11.5%	13.0%	0.479
Individuals reporting that enough time was spent with their provider during a previous visit	81.8%	76.0%	0.048*
Individuals who felt the provider took into consideration their values, beliefs, and traditions when recommending treatment	86.2%	70.0%	0.000*
Individuals reporting that they have health insurance	90.9%	91.0%	0.961

Table 2 shows the percentage of individuals reporting their total health care visit over the past year in this study as well as a previous study of the general U.S. population [5]. The ethnicity breakdown of the U.S. study is also given. Nepalese adults had less health care visits in the past year than the overall U.S. population.

**Table 2:** Health care visits in Nepalese adults and the general U.S. population U.S.: United States; difference in participant response and overall U.S. response is statistically significant (p<0.05)

Total Health Care Visits in the Past Year	Survey Participants	U.S.-Overall	U.S.-White	U.S.-Hispanic	U.S.-Black
No visits	16.9%	14.9%	15.2%	21.8%	14.8%
1 to 3 visits	70.2%	49.8%	49.6%	47.6%	52.1%
4 to 9 visits	7.4%	23.3%	23.3%	20.9%	22.8%
10+ visits	5.4%	11.9%	11.9%	9.6%	10.3%

**Figure 1:**
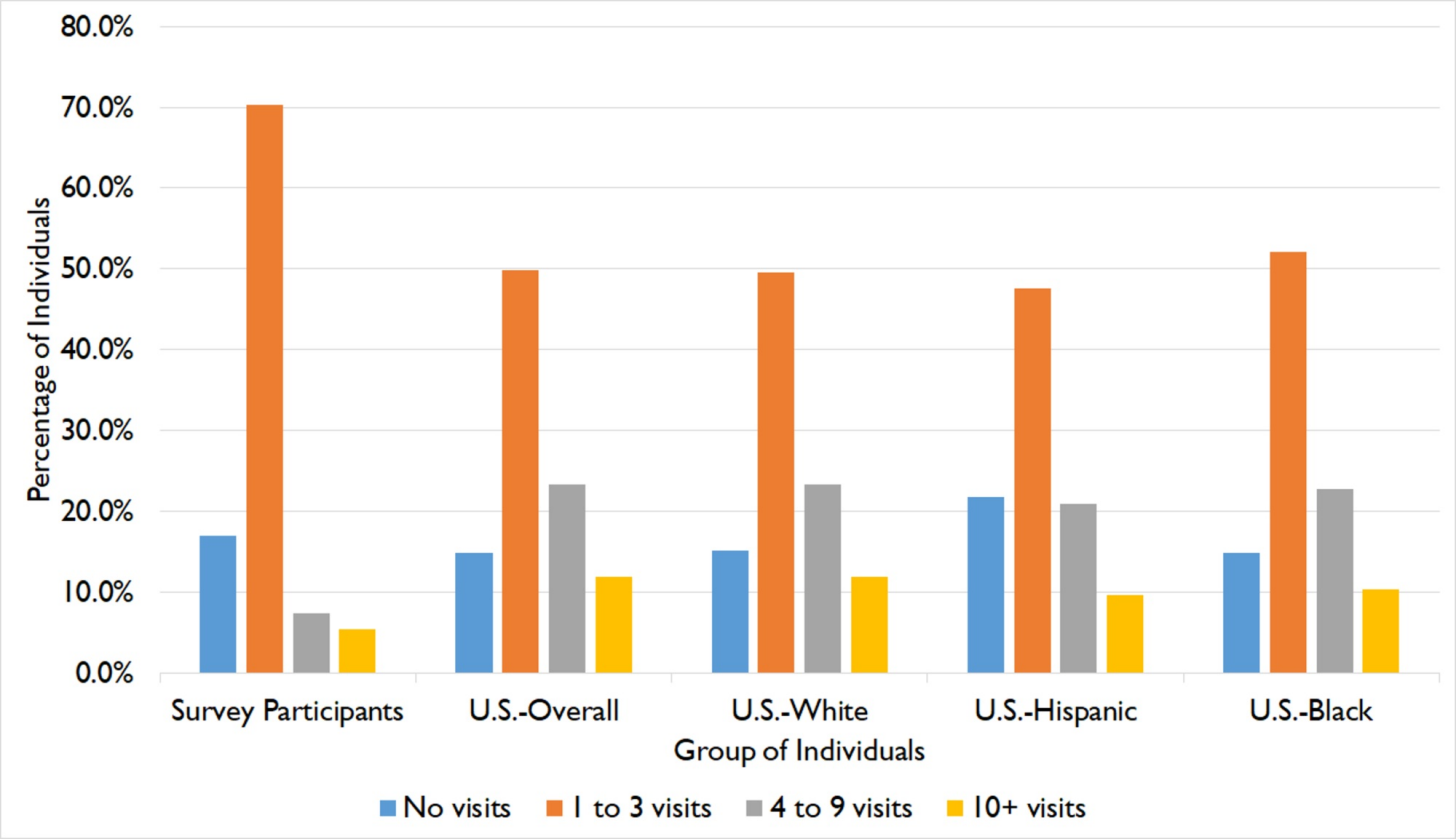
Health care visits in Nepalese adults and the general U.S. population U.S.: United States

The data in Table 2 are also presented in Figure 1 in graphical form. For each group of individuals, the percentage of the population that falls into each visit category (no visits, one to three visits, and so on) can be seen.

Table 3 shows the percentage of individuals who attended a preventative dental visit in the last year, 60.6% in the study vs. 71% in the U.S. population.

**Table 3:** Dental visit in Nepalese adults and the general U.S. population U.S.: United States; Asterisk (*): represents statistical significance (p<0.05)

	Participant Response	U.S. Statistics	p-value
Individuals who have been to a preventative dental visit in the past year	60.6%	71%	0.000*

Table 4 is a comparison of the percentage response by male and by female for study participants. Results are not statistically significant.

**Table 4:** Study participant response by gender

Topics	Male Response	Female Response
Individuals who had experienced a time they could not get the care needed in the past year	16.22%	25.19%
Individuals who felt language was a barrier to their care	9.82%	13.18%
Individuals reporting their health status as poor/fair	15.18%	8.33%
Individuals with primary care providers	91.51%	85.25%
Individuals who've been to preventative dental visit in past year	65.18%	56.59%
Total health care visits in the past year:		
No visits	13.39%	20.00%
1 to 3 visits	72.32%	68.46%
4 to 9 visits	8.04%	6.92%
10+ visits	6.25%	4.62%

 Table 5 is a comparison of percentage response by age group for study participants. Results are not statistically significant.

**Table 5:** Study participant response by age bracket

Topic	Age (≤24) year	Age (25-34) year	Age (35-44) year	Age (45-54) year	Age (≥55) year
Individuals who had experienced a time they could not get the care needed in the past year	19.0%	27.0%	15.3%	18%	36.8%
Individuals who had postponed getting the medical care needed in the past year	28.6%	21.9%	19.4%	9%	21.1%
Individuals with primary care providers	80.0%	81.7%	91.4%	94%	93.8%
Individuals who've been to a preventative dental visit in the past year	50.0%	48.4%	63.4%	69%	73.7%

## Discussion

Access limitations

When compared to the general Connecticut population, significantly more Nepalese participants reported that they had experienced a time they were unable to access necessary care within the past year (Table 1). Furthermore, when compared to national data, including specific racial/ethnic groups, Nepalese respondents reported significantly fewer total health care visits in the past year as well as significantly less preventative dental visits (Table 2 and Table 3). We speculate that this inability to access care as well as decreased health visits may be due to one or many of the following factors: cost of care or coverage by insurance, language barriers, cultural attitudes or approaches toward care, and difficulties in transportation.

The Institute of Medicine (IOM) has described three primary types of barriers to health access in the U.S.: structural barriers (the location and type of care available), financial barriers (health care coverage and ability to pay), and personal/cultural barriers (affects willingness and ability to seek care or follow treatment recommendations) [6]. Note that given the literature on health care barriers in U.S. Nepalese is substantially limited, the following speculations using the IOM categorization were made without such context. Structural barriers faced by Nepalese people may include the distance of the health care facility and lack of transportation, as well as the inability of facilities to accommodate this population (i.e., limited language services and provider discomfort in effectively treating specific populations). As for financial barriers, although this study’s participants had a similar proportion of health care coverage to the general CT population (Table 1), the following questions still exist: do CT Nepalese generally have limited income, resulting in a difficulty paying for health care? Does this group tend to have coverage types that limit the individuals from accessing necessary care? Answers to these questions will be necessary before determining the role that financial barriers play in this population’s care.

We believe that personal/cultural factors are the major barriers to access in this population. Participants may feel uncomfortable approaching providers for help or, conversely, feel that they are generally healthy and choose to avoid care. Previous literature describes religion (belief of what illness is and why it has occurred) and tradition (i.e. use of herbal therapies and home remedies and therapeutic rituals) as factors that may influence the patient's understanding of health-related situations and their willingness to seek care [7-8]. It is well-known that in Nepal, alternative practices, such as Ayurveda, Tibetan medicine, homeopathy, and faith healing, continue to exist in addition to Western medicine [9]. In fact, faith healing seems to be the most prevalent medical system in Nepal. Unfortunately, this practice is not centralized, lacks the codification of knowledge and the professional organization of healers, and varies by village and by individual. Nepalese may avoid seeking traditional care for illnesses and defer to spiritual leaders for treatment instead. This is supported by one study, which examined the use of diabetic treatments in Nepalese with findings suggestive of reluctance in taking conventional antidiabetic medication due to a preference for utilizing natural alternatives that they perceived to be healthier [10]. Additionally, preventative health services and health promotion measures are limited in the country of Nepal [11]. In addition to the lack of health workers and government resources, there seems to be a lack of interest in preventive and promotive medicine. For these reasons, individuals in Nepal generally do not take initiatives such as immunization, regular doctor visits, or healthy behavioral changes. It is likely that such cultural factors described in Nepal are also true for the Nepalese in the U.S. The health access limitations seen in our population may be a result of individual beliefs regarding alternative medicine as well as a disinterest in preventative care.

The responses in this study regarding health access raise many questions of “why.” We have speculated on causes, but future studies should be done to examine the specific barriers while keeping in mind the factors that can be addressed to ultimately improve care in the Nepalese population.

Perception of care

One surprising finding was that more Nepalese felt that providers spent enough time with them (81.8% vs 76%) during previous visits and that their values, beliefs, and traditions were taken into consideration (86.2% vs 70%) during treatment recommendation as compared to the general CT population. Despite the access limitations and decreased visits described earlier, survey respondents felt generally more satisfied with their visits and interactions with providers.

We speculate that personal/cultural perceptions of health and healthcare may play a role. Nepalese individuals may feel generally healthier than other ethnic groups, resulting in overall satisfaction with their health and health care interactions. Additionally, previous studies have demonstrated that patients who perceive providers as culturally competent tend to be more compliant with treatment recommendations [12]. Nepalese in CT may feel content with their provider’s cultural competency, resulting in subsequent acceptance of treatment plans and satisfaction of care.

In answering the question of why CT Nepalese are more satisfied in their interactions with providers, it is important to consider cultural norms relating to how Nepalese generally perceive physicians. In many countries, medical paternalism continues to determine patient-healer interactions. One study examining patient-centered communication in Nepal demonstrated that the patient-healer decision-making partnership in Nepal is unequal and that individuals were satisfied with doctors taking control of decisions regarding health [13]. It is possible that this perception may hold true for Nepalese in the U.S. as well. These individuals may be more willing to let providers take control of interactions and recommendations, resulting in overall satisfaction with service.

Study limitations and future studies

This study had several notable limitations. The sample size was relatively small (n=244) and, therefore, did not allow for detecting smaller but potentially important differences. For example, we did not identify statistically significant differences between males and females or between age groups (Table 4 and Table 5). However, this is not to say that differences do not exist. Understanding gender and age disparities in health access/utilization can allow for more efficient and individualized approaches to improving care. There is room for future research of these intrapopulation subgroups likely in a larger sample size.

Another weakness of the study was its recruitment method. Respondents of the survey were recruited from only two major Nepalese gatherings in two different cities in Connecticut. Therefore, the results may not be representative of the greater Nepalese population in the state, as they may not reflect the health care behaviors and perceptions of those who did not attend these events or those who live in parts of the state that are far from these locations. Factors confounding the results of the access/utilization survey must be considered. For example, because these respondents had access to transportation to come to the event, they may also be more likely to have access to transportation to health care facilities than CT Nepalese who did not attend the event. Follow-up studies examining this population should consider alternative recruitment methods, one that includes the scope of Nepalese participants in most or all parts of the state.

The population examined was only of Nepalese in CT and may not be generalizable to Nepalese in other states, as health policy and law vary state by state. Follow-up studies can examine not only the specific factors affecting access and the perception of care, but they can also ask similar questions in Nepalese populations of other states.

## Conclusions

In conclusion, this study explored health access and utilization in the Connecticut Nepalese adult population with findings significant for health access limitations but overall satisfaction with care. Several speculations were made regarding potential structural, financial, and, most importantly, cultural factors relating to these findings. These conclusions provide a good foundation for future work in this rapidly growing immigrant population, both at the research and public health initiative levels.
